# Si-Jun-Zi Decoction Treatment Promotes the Restoration of Intestinal Function after Obstruction by Regulating Intestinal Homeostasis

**DOI:** 10.1155/2014/928579

**Published:** 2014-04-28

**Authors:** Xiangyang Yu, Zhigang Cui, Zhenli Zhou, Tao Shan, Donghua Li, Naiqiang Cui

**Affiliations:** ^1^Department of Gastrointestinal Surgery, Nankai Hospital, Nankai District, Tianjin 300100, China; ^2^Graduate School of Tianjin Medical University, Nankai District, Tianjin 300100, China; ^3^Tianjin Institute of Acute Abdominal Disease of Integrated Traditional Chinese and Western Medicine, Nankai District, Tianjin 300100, China; ^4^Department of Hepatopancreatobiliary Surgery, Nankai Hospital, Nankai District, Tianjin 300100, China

## Abstract

Intestinal obstruction is a common disease requiring abdominal surgery with significant morbidity and mortality. Currently, an effective medical treatment for obstruction, other than surgical resection or decompression, does not exist. Si-Jun-Zi Decoction is a famous Chinese medicine used to replenish qi and invigorate the functions of the spleen. Modern pharmacological studies show that this prescription can improve gastrointestinal function and strengthen immune function. In this study, we investigated the effects of a famous Chinese herbal formula, Si-Jun-Zi Decoction, on the restoration of intestinal function after the relief of obstruction in a rabbit model. We found that Si-Jun-Zi Decoction could reduce intestinal mucosal injury while promoting the recovery of the small intestine. Further, Si-Jun-Zi Decoction could regulate the intestinal immune system. Our results suggest that Si-Jun-Zi Decoction promotes the restoration of intestinal function after obstruction by regulating intestinal homeostasis. Our observations indicate that Si-Jun-Zi Decoction is potentially a therapeutic drug for intestinal obstruction.

## 1. Introduction

Intestinal obstruction is a common disease requiring abdominal surgery with significant morbidity and mortality. Intestinal obstruction often occurs in the small or large intestines [1]. Regardless of the initial cause of the obstruction, a series of pathophysiological changes occur in the obstructed segments. These changes are responsible for symptoms such as bloating, vomiting, abdominal cramps, and constipation and may lead to intestinal failure [2].

The function of the intestine rests on the normal and balanced homeostasis of the intestine, while intestinal homeostasis depends upon complex interactions between the intestinal epithelium and the intestinal immune system [3]. On a cellular level, the dynamic crosstalk between intestinal epithelial cells (IECs) and local immune cells represents one of the fundamental features of intestinal homeostasis. These interactions are not only important for the pathogenesis of intestinal disorders such as IBD, Crohn's disease, and intestinal obstruction, but also essential for maintaining normal intestinal homeostasis [4]. Our previous study has shown that intestinal obstruction can induce severe dysfunction of intestinal homeostasis. The intestinal epithelial cells and the intestinal immune system are significantly compromised as the obstruction progresses. Therefore, restoration of intestinal homeostasis may be an attractive strategy for treatment of human intestinal obstruction.

The Chinese medicine Si-Jun-Zi Decoction is a famous herbal formula composed of four Chinese herbs: Ginseng Root,* Atractylodes macrocephala*, licorice root, and Poria root. This formula is considered mild in nature. It balances qi and invigorates the spleen. Modern pharmacological studies show that this prescription affects several other physiological functions such as improving gastrointestinal function [5], strengthening the immune system [6, 7], improving bone marrow hematopoietic function, and speeding up the production of red blood cells. This prescription can also be used for the treatment of malignant tumors of the digestive tract [8]. Xiao and Yang found that Si-Jun-Zi Decoction could improve the immune function and quality of life and reduce the side effects of chemotherapy in patients with colorectal cancer undergoing chemotherapy [7]. Liu et al. found that Si-Jun-Zi Decoction could promote the recovery of glucose uptake in the small intestine of reserpine induced Pi-qi deficiency syndrome rats [9]. Modified Si-Jun-Zi Decoction could slow down the formation of prednisone-induced osteoporosis through promoting osteoblast differentiation and inhibiting osteoclastogenesis [10]. A clinical observation by Guo et al. found that Chenxia Si-Jun-Zi Decoction could promote severe patient's gastrointestinal function recovery and reduce hospitalization days [5]. However, the therapeutic effects of Si-Jun-Zi Decoction on cases of intestinal obstruction are unknown.

In the present study, we investigate the effects of Si-Jun-Zi Decoction on the restoration of intestinal function after the relief of obstruction in a rabbit model. Our results indicate that Si-Jun-Zi Decoction promotes the restoration of intestinal function by regulating intestinal homeostasis. Our observations indicate that Si-Jun-Zi Decoction may have potential therapeutic effects on patients suffering from intestinal obstruction.

## 2. Materials and Methods

### 2.1. Animals and Reagents

Healthy New Zealand rabbits with a body weight of 2.5–3 kg were used in this study. The animals were purchased from Mingle Laboratory Animal Center (Tianjin, China) and maintained in a temperature-controlled room with a 12 h light/dark cycle and with access to regular chow and water. The experimental procedures were approved by the Laboratory Animal Care Committee at Tianjin Medical University. All animals received care according to the Guide for the Care and Use of Laboratory Animals (NIH, Bethesda, MD). The Si-Jun-Zi Decoction was supplied by the pharmaceutical preparation section of Nankai Hospital (Tianjin, China). The formula was composed of tuckahoe root (*Scierotium poriae cocos*), ginseng root (*Radix ginseng*), white* Atractylodes* rhizome root (*Rhizoma Atractylodes macrocephalae*), and licorice root (*Radix glycyrrhizae*) in the ratio of 3 : 3 : 2 : 2 (tuckahoe root 60 g, ginseng root 60 g, rhizome root 60 g, and licorice root 40 g), which were submerged in 2.2 L distilled water for 30 min and then decocted twice (2 h per time), then filtered and concentrated to 1 g/mL using a routine method, and stored in a refrigerator at 4°C until use.

### 2.2. Description of Experimental Groups and the Rabbit Intestinal Obstruction Model

Thirty-six New Zealand rabbits were randomly divided into six groups: sham operation control group (Sham), 48 h after obstruction group (O_48 h_), natural recovery at 48 h or 96 h after relief of the obstruction groups (S_48 h_, S_96 h_), and Si-Jun-Zi Decoction treatment for 48 h or 96 h after the relief of the obstruction groups (T_48 h_, T_96 h_), (*n* = 6 per group). To establish a rabbit model of intestinal obstruction that was controllable, we transformed the parts of infusion sets that are widely used in clinical applications into an* in vitro* pulled-type lock. After being anesthetized with an i.v. injection of urethane (1 g/kg), a laparotomy was performed on sedated rabbits and a uniform controllable loop obstruction was created in the mesenteric nonavascular zone by placing a clamp eight cm from the distal end of the ileum. Sham-operated rabbits received mock manipulation of the gut without placement of the lock. The animals were allowed to recover postoperatively for 3 days to remove any influence of the anesthesia on our test parameters. Three days after the operation, the clamp was locked according to the color label, resulting in the obstruction of the intestine. The obstruction lasted for 48 h after which the intestinal obstruction was relieved by cutting off the lock. The animals were then treated with or without drug (5 g/kg, twice a day) for 48 h or 96 h. At the end of the experiment, the animals were sacrificed for further analysis.

### 2.3. Quantification of Intestinal Damage and Determination of Intraepithelial Lymphocytes (IEL) and Lamina Propria Lymphocytes (LPL) Cell Numbers

Two-centimeter or longer intestinal segments from the ileum (5 cm from the distal ileum with obstruction) were excised, fixed in 4% paraformaldehyde, and embedded in paraffin blocks. Four-micron thick sections were stained with H&E and PAS using standard protocols [11]. The sections were examined using a Champion-500w graphic report management system. Intestinal mucous membrane damage was evaluated and Chiu's [12] histopathological scores were determined. Briefly, the tissue damage was graded from 0 to 5 according to the following criteria: grade 0, normal structure of villi; grade 1, development of small subepithelial space at the villous apex; grade 2, enlarged subepithelial space but without change in villous length and width; grade 3, few shortened villi and presence of cells in the lumen; grade 4, the majority of villi are shortened and widened with crypt hyperplasia and cells in the lumen; and grade 5, blunting of all villi with elongated crypts and a large number of cells in the lumen. The percentage of intraepithelial lymphocytes (IELs) and lamina propria lymphocytes (LPLs) were quantified in photomicrographs of the intestines. The percentage of IEL cells was calculated using the following formula: (the number of total IEL cells in 20 villi per section/the total cell number in 25 villi per section) × 100. The percentage of LPL cells was calculated using the following formula: (the number of total LPL cells in 20 villi per section/the total lamina propria cell number in the central axis of 25 villi per section) × 100.

### 2.4. High-Performance Liquid Chromatography (HPLC) Analysis of Ornithine Decarboxylase Activity and Citrulline Levels

Theornithine decarboxylase (ODC) activity of intestinal tissues and the level of citrulline in blood serum were analyzed by HPLC as described elsewhere [13, 14].

### 2.5. RNA Extraction and Real-Time PCR

RNA was extracted from intestinal segments (100 mg) from the ileum (5 cm from the distal ileum with obstruction) using a Total RNA Kit (Qiagen) by following the manufacturer's instructions. First-strand cDNA was synthesized from 1 *μ*g mRNA using reverse transcriptase (Fermentas, Glen Burnie, MD) and oligo (dT) primers. The primer sequences used were as follows: Claudin 1 forward: 5′-GTGCCTTGATGGTGATTG-3′, reverse: 5′-AAAGTAGCCAGACCTGAAAT-3′. *β*-actin forward: 5′-TGATGGTGGGCATGGGTC-3′, reverse: 5′-CGATGGGGTACTTCAGGGTG-3′. Real-time PCR was performed using an Applied Biosystems PRISM7300 (Applied Biosystems) and SYBR Green PCR master mix (Applied Biosystems). Reagents concentration were 2× Realtime Mix 10 *μ*L, forward primer 0.3 *μ*L, reverse primer 0.3 *μ*L, ddH_2_O 7.4 *μ*L, and cDNA templates 2 *μ*L. PCR cycling conditions were 95°C for 30 sec followed by 40 cycles of 95°C for 5 sec and 50°C for 30 sec. The mRNA expression was normalized to the expression of the *β*-actin housekeeping gene.

### 2.6. Flow Cytometry Analysis of Peyer's Patch (PP) Lymphocytes

The PP lymphocytes were isolated as described previously [15]. Briefly, the excised PP lymphocytes were incubated under sterile conditions with RPMI medium containing 1 mM DTT (5 min, 37°C). Thereafter, the PP lymphocytes were washed with RPMI medium and passed through a steel mesh. The resulting cell suspension was washed and resuspended in RPMI containing 10% FBS. The PP lymphocytes were stained with FITC-anti-rabbit CD4, PE-anti-rabbit CD8, and Percp-Cy5.5-anti-rabbit CD3. A negative control was stained with isotype-matched mAb. Cells were stained at 4°C for 30 min. The cells were then washed and resuspended in PBS for FACS analysis. Data were acquired using a FACSCalibur (BD Bioscience) flow cytometer and analyzed using the CellQuest software.

### 2.7. ELISA

A fresh 8 cm intestinal segment from the ileum (5 cm from the distal ileum with obstruction) was obtained, the intestinal solid contents were removed, and casing slime was collected by douching with 3 mL of sterile PBS. The obtained extracts were centrifuged and the supernatants were collected. The levels of s-IgA were measured using an ELISA kit according to the manufacturer's instructions. The serum level of D-lactate was measured by ELISA kit (ADL co.), according to the manufacturer's instructions.

### 2.8. Statistical Analysis

Results were expressed as mean ± SD. The data were analyzed using a two-tailed Student's *t*-test (GraphPad Prism 5) and a *P* value of <0.05 was regarded as statistically significant.

## 3. Results

### 3.1. The Si-Jun-Zi Decoction Reduces Intestinal Mucosal Injury

**Figure 1:**
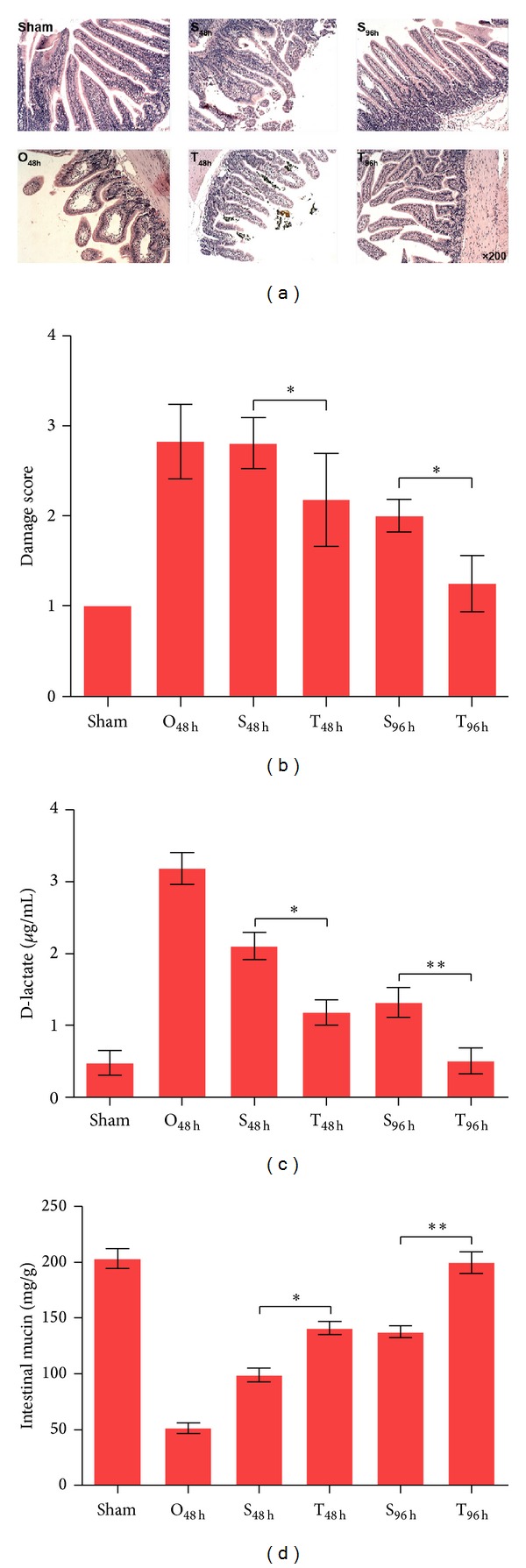
Si-Jun-Zi Decoction treatment reduces intestinal mucosal injury. (a) and (b): intestinal segments from the ileum were excised and fixed in 4% paraformaldehyde and embedded in paraffin blocks. We stained 4 *μ*m thick sections with H&E. Intestinal mucous membrane damage and Chiu's histopathological score were evaluated. The H&E staining (a) and the damage score (b) are shown. In panel (c), the serum level of D-lactate is shown. In panel (d), the level of intestinal mucins, examined by Coomassie brilliant blue G250 staining, is shown. The data are representative of three independent experiments, with each using 6 mice per group. Data are shown as mean ± SD. (*P < 0.05, **P < 0.01).

The intestinal mucosal barrier is the first line of host defense against intestinal pathogens, and intestinal obstruction induces sever intestinal mucosal injury [16]. Intestinal mucosal injury is also a common clinical complication that may lead to dysfunction of the intestinal barrier. Here, by using a modified and controllable rabbit intestinal obstruction model, we evaluated the ability of the Si-Jun-Zi Decoction to reduce the obstruction-induced intestinal mucosal damage after relief of the intestinal obstruction. As shown in Figures 1(a) and 1(b), the intestinal obstruction induced severe intestinal mucosal injury, including villous blunting and epithelial sloughing. The overall histopathological damage score of the intestines of the sham and O_48 h_ groups was statistically different. After relieving the obstruction, the damage to the intestinal mucosa was gradually repaired, while the Si-Jun-Zi Decoction treatment groups showed significantly increased levels of repair compared to treatment groups without Si-Jun-Zi Decoction treatment. The overall histopathological damage score of the S_48 h_ versus T_48 h_ groups and the S_96 h_ versus T_96 h_ groups was statistically different (*P* < 0.05), suggesting that the Si-Jun-Zi Decoction treatment had a profound protective effect against intestinal mucosal injury. This protection provided by the Si-Jun-Zi Decoction was confirmed by changes in the level of blood D-lactate, which is a measure of intestinal mucosa permeability [17], and the intestinal mucins, which are key components of the intestinal mucosal barrier [18]. The data showed that D-lactate levels significantly declined after Si-Jun-Zi Decoction treatment (S_48 h_ versus T_48 h_ and S_96 h_ versus T_96 h_) (Figure 1(c)), while levels of the intestinal mucins significantly increased relative to the control groups (S_48 h_ versus T_48 h_ and S_96 h_ versus T_96 h_) (Figure 1(d)). These results indicate that the Si-Jun-Zi Decoction could reduce the permeability of intestinal mucosa and enhance the secretion of intestinal mucins. Taken together, our results showed that the Si-Jun-Zi Decoction was beneficial for the reduction of intestinal mucosal injury after relieving the intestinal obstruction.

### 3.2. The Si-Jun-Zi Decoction Promotes the Recovery of the Small Intestine

**Figure 2:**
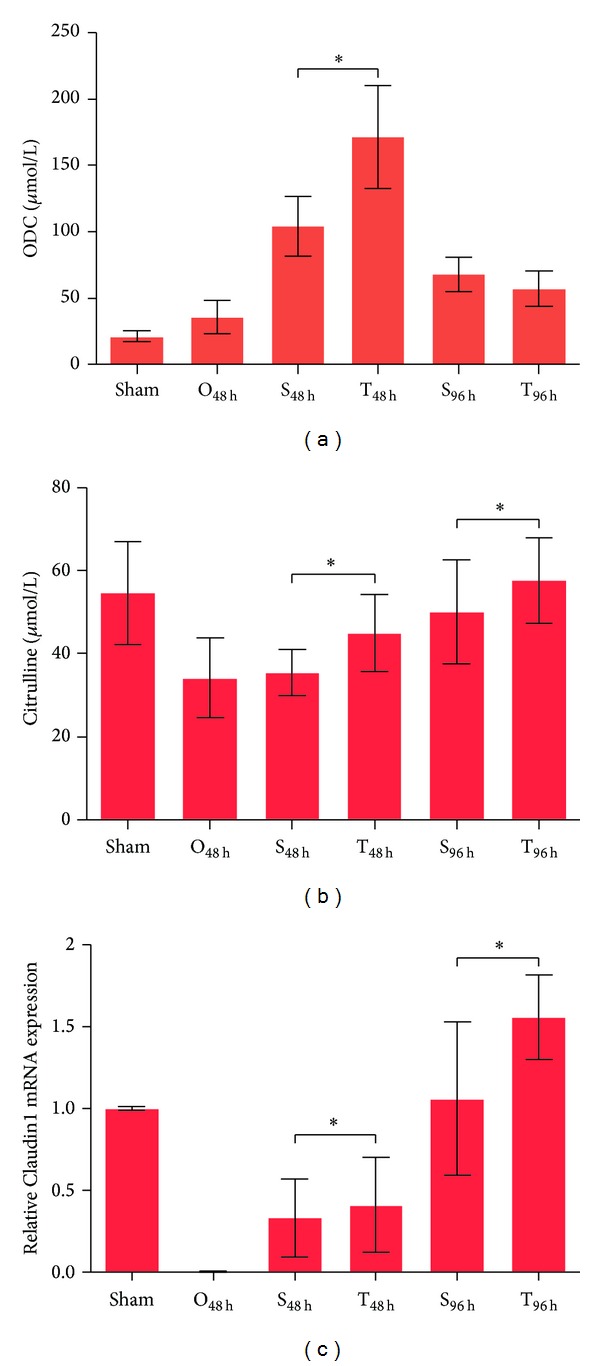
Si-Jun-Zi Decoction treatment promotes recovery of the small intestine. (a) and (b): the ODC activity (a) in intestinal tissues and the level of citrulline (b) in blood serum were analyzed by HPLC. The data are shown as mean ± SD (*n* = 6). (c) The gene expression level of Claudin 1 was examined by qRT-PCR. The data are shown as the mean ± SD (*n* = 6). The data are representative of at least three independent experiments. *P < 0.05.

We next evaluated the effects of the Si-Jun-Zi Decoction on the function and integrity of the small intestine. We first examined the change in ornithine decarboxylase (ODC) levels, which indicate intestinal epithelial cells proliferation [19]. Our previous study showed that the level of ODC increased after obstruction, reaching a peak at 12 h after obstruction, and then rapidly decreased. This observation suggests that, during the early stages of obstruction, the intestinal epithelial cells renewed quickly, promoting the recovery of the intestinal epithelium, and that the damage and recovery were balanced. However, at later stages, this balance was upset due to the severity of intestinal epithelial damage. As shown in Figure 2(a), the ODC level of the O_48 h_ group was equal to the sham control group and gradually increased after the obstruction was relieved. The level of ODC was significantly elevated after 48 h of Si-Jun-Zi Decoction compared with the S_48 h_ group. At 96 h after relieving the obstruction, the ODC level returned to normal and thus maintained intestinal homeostasis. This result indicates that the Si-Jun-Zi Decoction could promote the recovery of the intestinal epithelium. We next quantified the level of blood citrulline, which serves as an indicator of the absorptive function of small intestine [20]. Our data showed that serum levels of citrulline in the T_48 h_ or T_96 h_ groups were significantly higher than those in the S_48 h_ or S_96 h_ groups (Figure 2(b)), suggesting that the Si-Jun-Zi Decoction treatment promoted the recovery of the absorptive function of the small intestine. Because the integrity of the intestinal mucosa, which is maintained by tight junction proteins (TJPs) and adherens junction proteins, is essential for the function of the intestinal barrier [21], we examined Claudin 1 gene expression by real-time PCR analysis. Claudin 1 is the functional component of tight junction transmembrane protein (TJPs) in intestinal epithelial cells [22]. We found almost undetectable levels of Claudin 1 gene expression at 48 h after obstruction, indicating that the intestinal epithelial integrity at later stages of obstruction was completely lost (Figure 2(c)). Once the obstruction was removed, Claudin 1 gene expression increased (S_48 h_ group). In animals treated with the Si-Jun-Zi Decoction for 48 h, expression of the Claudin 1 gene was significantly increased compared with the S_48 h_ group, and this enhancement was more obvious in T_96 h_ group versus T_48 h_ group. This result indicates that the Si-Jun-Zi Decoction could promote the recovery of the integrity of the small intestine. Taken together, our results suggest that the Si-Jun-Zi Decoction can promote the recovery of the small intestine after relieving intestinal obstruction.

### 3.3. The Si-Jun-Zi Decoction Regulates the Intestinal Immune System

**Figure 3:**
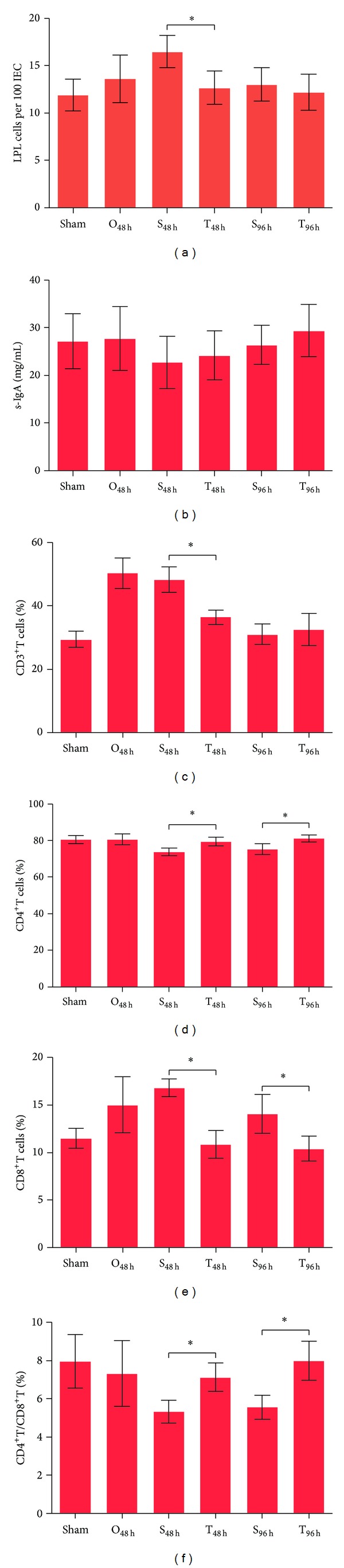
Si-Jun-Zi Decoction treatment regulates the intestinal immune system. (a) Intestinal segments from the ileum were excised and fixed in 4% paraformaldehyde and embedded in paraffin blocks. We stained 4 *μ*m thick sections with PAS, and the percentage of LPLs was quantified in photomicrographs of the intestines. (b) The level of s-IgA in the intestinal lumen was examined by ELISA. (c)–(f) The PP lymphocytes were isolated and the percentage of T cells subtypes was analyzed by flow cytometry. The percentage of CD3^+^T cells (c), the percentage of CD4^+^T cells (d), the percentage of CD8^+^T cells (e), and the CD4^+^/CD8^+^T cells ratio (f) are shown. The data are representative of three independent experiments, with each using 4-5 mice per group. Data are shown as mean ± SD (*P < 0.05).

Intestinal epithelial cells are well known for their role as the boundary between the external environment and the intestinal tract. Apart from being a physical barrier, intestinal epithelial cells play a pivotal role in regulating immune responses in order to maintain intestinal homeostasis [23]. However, once intestinal epithelial cell damage occurs, the balance between intestinal epithelial cell barrier and intestinal immune system cannot be maintained. Our previous study found that intestinal obstruction induces intestinal immune system dysfunction and therefore we investigated whether the Si-Jun-Zi Decoction could regulate the intestinal immune system after intestinal obstruction was relieved. We first assessed the number of intraepithelial lymphocytes (IELs) and lamina propria lymphocytes (LPLs) and intestinal epithelial cells (IEC) using PAS stained tissue sections. The data showed that significant differences in the numbers of IELs after Si-Jun-Zi Decoction treatment (data not shown) were not found, while the number of LPLs decreased with the Si-Jun-Zi Decoction treatment (S_48 h_ versus T_48 h_, *P* < 0.05) (Figure 3(a)). In addition, we analyzed the level of s-IgA in the intestinal lumen at various times, with or without drug treatment, after relieving the obstruction. We found that the s-IgA level was not significantly changed, suggesting that the Si-Jun-Zi Decoction does not affect the innate immunity of the intestine. We next examined alterations in T cells subtype percentages in PP lymphocytes by flow cytometry. As shown in Figures 3(c)–3(f), Si-Jun-Zi Decoction treatment could reduce the percentage of CD3^+^T cells 48 h after relieving the intestinal obstruction compared with the no treatment group (Figure 3(c)). Si-Jun-Zi Decoction treatment could increase the percentage of CD4^+^T cells and reduce the percentage of CD8^+^T cells at different times with or without drug treatment after the relief of obstruction (Figures 3(d) and 3(e)). The CD4^+^/CD8^+^T cells ratio was also sharply elevated by Si-Jun-Zi Decoction treatment (Figure 3(f)). These data suggest that the Si-Jun-Zi Decoction could regulate the adaptive immune response by reducing the number of CD3^+^T cells and CD8^+^T cells while increasing the number of CD4^+^T cells. However, the precise mechanisms involved still require further investigation. Based on the above data, we suggest that the Si-Jun-Zi Decoction can promote the restoration of intestinal function by regulating the intestinal immune system.

## 4. Discussion

Intestinal obstruction is a common disease requiring abdominal surgery with significant morbidity and mortality [1]. The major morbidity associated with intestinal obstruction is related to excessive distension that leads to strangulation and bowel necrosis. Until now, effective medical treatment for obstruction, other than surgical resection or decompression, is lacking. However, even if the obstruction is surgically removed, many patients continue to have disturbed motility function in the bowel proximal to the site of resection for many years to come [24, 25]. There are very few drugs for the treatment of intestinal obstructions. Recently Alvimopan, a drug that behaves as a peripherally acting *μ*-opioid receptor antagonist, was approved by the FDA for the treatment of patients with postoperative ileus (POI) following partial large or small bowel resection surgery with primary anastomosis [26]. However, there are several side effects and limitations for the use of this drug [27, 28].

Si-Jun-Zi Decoction is a famous Chinese medicine used to replenish qi and invigorate the functions of the spleen. It is used when there is a deficiency of qi of the spleen and stomach, which is marked by anorexia and loose bowels. Modern pharmacological studies show that this prescription can improve gastrointestinal function [5] and strengthen immune function [6, 7]. Therefore, we hypothesized that this prescription may be beneficial for the restoration of intestinal function after relieving the obstruction.

In the current study, we used a controllable rabbit intestinal obstruction model to investigate the effects of a Si-Jun-Zi Decoction on the restoration of intestinal function after relieving the obstruction. We found that the Si-Jun-Zi Decoction can reduce the intestinal mucosal injury. Si-Jun-Zi Decoction treatment significantly alleviated intestinal mucosal damage, reduced intestinal mucosal permeability, and enhanced the secretion of intestinal mucins. Furthermore, the Si-Jun-Zi Decoction promotes the recovery of the small intestines. Si-Jun-Zi Decoction treatment could promote the recovery of both the intestinal epithelium and the absorptive function of the small intestine by increasing ODC and citrulline levels. In addition, the Si-Jun-Zi Decoction promotes the recovery of small intestine integrity by upregulating the Claudin 1 gene expression. Finally, we found that the Si-Jun-Zi Decoction regulates the intestinal immune system. Although the Si-Jun-Zi Decoction does not affect the innate immunity of the intestine, it may regulate the adaptive immune response by reducing CD3^+^T cell and CD8^+^T cell numbers while increasing the number of CD4^+^T cells in PP lymphocytes.

Although we found that the famous Chinese herbal formula Si-Jun-Zi Decoction has the ability to promote the restoration of intestinal function by regulating the intestinal homeostasis, the precise mechanisms involved are still to be elucidated. Si-Jun-Zi Decoction as a potential therapeutic drug for intestinal obstruction treatment needs to be investigated in the future.

## 5. Conclusion

The present study clearly demonstrates that Si-Jun-Zi Decoction promotes the restoration of intestinal function after obstruction by regulating intestinal homeostasis. Our results indicate that the Si-Jun-Zi Decoction may be a potential therapeutic drug for intestinal obstruction. Further studies are needed to assess the side effects and limitations of this drug in the treatment of intestinal obstruction.
